# Polymer models of chromatin organization in virally infected cells

**DOI:** 10.1042/BST20240598

**Published:** 2025-02-07

**Authors:** Andrea Fontana, Fabrizio Tafuri, Alex Abraham, Simona Bianco, Andrea Esposito, Mattia Conte, Francesca Vercellone, Florinda Di Pierno, Sougata Guha, Ciro Di Carluccio, Andrea M. Chiariello

**Affiliations:** 1 Dipartimento di Fisica, Università degli Studi di Napoli Federico II, Complesso Universitario di Monte Sant’Angelo, 80126 Naples, Italy; 2 INFN Napoli, Complesso Universitario di Monte Sant’Angelo, 80126 Naples, Italy; 3 Dipartimento di Ingegneria Chimica dei Materiali e della Produzione Industriale - DICMaPI, Università degli Studi di Napoli Federico II, Piazzale Vincenzo Tecchio 80, 80125 Naples, Italy

**Keywords:** Polymer physics, Molecular dynamics, Chromatin organization, Viral infections, SARS-CoV-2 virus

## Abstract

Genome architecture is closely tied to essential biological functions, yet a complete understanding of the mechanisms governing DNA folding remains a significant challenge. Theoretical models based on polymer physics have been applied to decipher the complexity of chromatin architecture and uncover the physical processes shaping its structure. Importantly, recent findings suggest that certain viruses can alter the 3D organization of the host genome. In this review, we highlight recent advances in the development of polymer models used to study how chromatin 3D structure within a cell re-organizes following viral infection, with a particular emphasis on the SARS-CoV-2 virus, capable of altering genome organization of the host cell at different scales, including A/B compartments, TADs and gene-enhancer regulatory contacts.

## Introduction

A great deal of research has been undertaken on SARS-CoV-2 viral infection, including the analysis of the immunological response to the virus [1] and the exploration of the effects of infection on epigenetic regulation of the host cells [2]. It has been discovered, for instance, that the virus affects the activity of key gene categories essential for the immune response [1], such as genes involved in the interferon (IFN) response and pro-inflammatory genes. Furthermore, recent studies have revealed that some viral infections can re-arrange chromatin architecture of the infected cell [3]. SARS-CoV-2, for instance, has been shown to induce alterations of chromatin architecture in the olfactory receptors [4] of humans and hamsters and general re-arrangements on multiple structural scales, from A/B compartments to topologically associated domains (TADs) [5]. On the other hand, polymer physics models have been shown to be a general and powerful tool to investigate chromatin organization [6–8].

In this review, we focus on recent applications of polymer physics models [9] that, through extensive molecular dynamics (MD) simulations, have been effective in quantitatively analyzing chromatin re-arrangements induced by SARS-CoV-2 viral infection in host cells and observed in experimental data [5]. On scales of several Mbp, a block copolymer model [10,11] is able to explain the observed weakening of A-compartments and increased A–B mixing in the SARS-CoV-2-infected genome. At the TAD level, a model combining loop-extrusion (LE) [12,13] and phase-separation (PS) [11,12] mechanisms describes the observed weakening of intra-TAD interactions in SARS-CoV-2-infected cells. In addition, combined with Hi-C data [14,15], the model recapitulates the architectural changes of genomic loci containing the *DDX58* and *IFIT* genes, which are linked to the IFN response [16] of the host cells. Interestingly, the model also provides testable predictions of structural changes, as analysis of polymer configurations shows that, in the SARS-CoV-2-infected condition, the variability of single-molecule 3D configurations is increased compared with non-infected conditions. This suggests that the observed changes in the activity of IFN genes [1,5] may be due to a general loss of structural specificity, resulting from alterations in the physical mechanisms governing 3D chromatin organization [17].

Finally, the model indicates that these changes in genomic organization due to SARS-CoV-2 infection result from a combined alteration of LE and chromatin PS properties, providing a physical mechanism potentially linking the observed genome re-structuring and the mis-regulation of crucial genes involved in the immune response [9].

### Polymer physics models to study chromatin architecture

To understand the complexity of chromatin organization and the molecular principles shaping it, different mechanisms have been proposed, supported by increasing experimental evidence, and incorporated in polymer models that are typically studied using numerical simulations [6]. In this section, we briefly review the main features of these models and the emerging understanding of the physical forces driving chromatin spatial organization.

**Figure 1:**
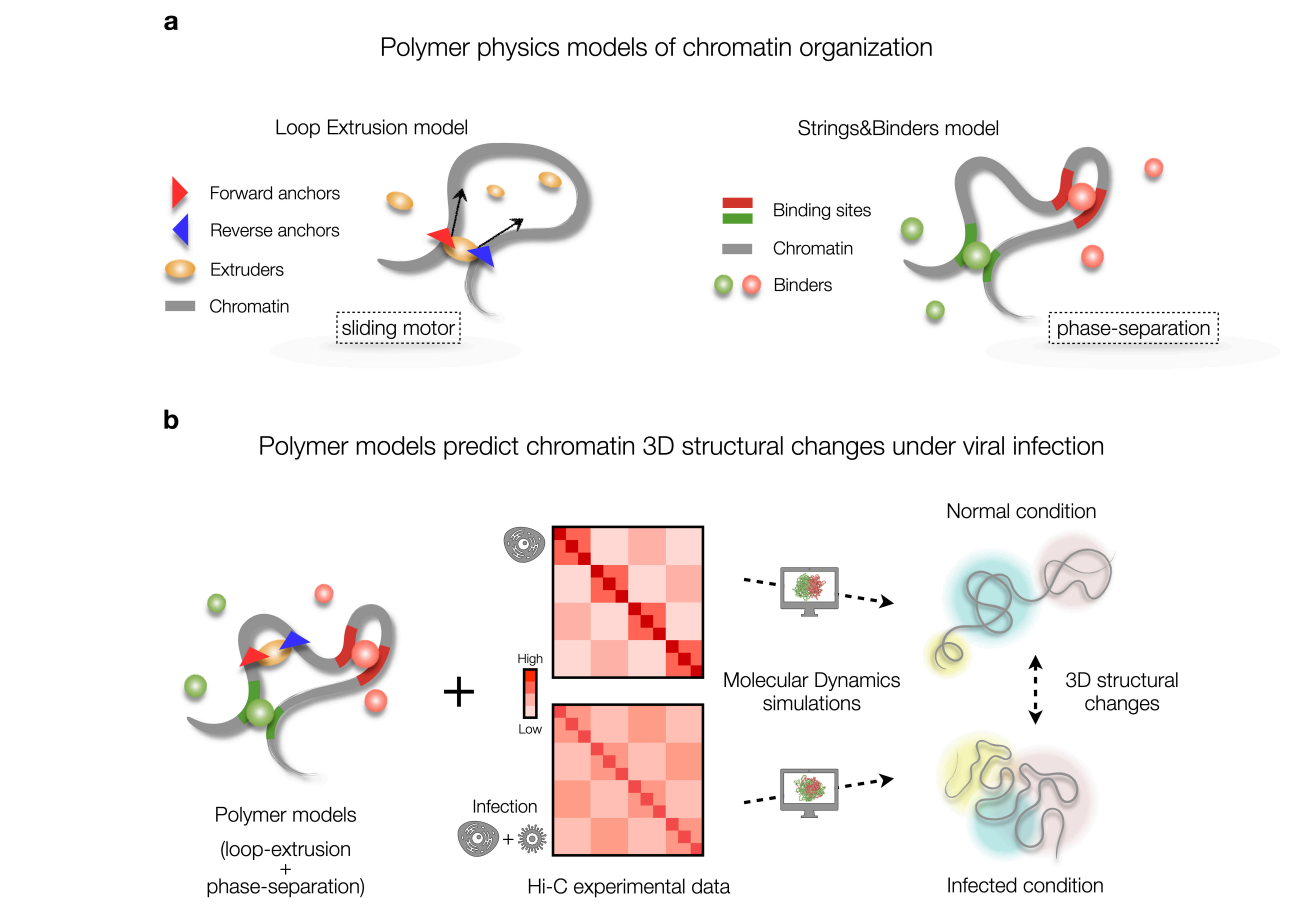
Polymer physics models of chromatin spatial organization and chromatin re-arrangement driven by viral infection. **(a)** Physical mechanisms shaping chromatin structure. The loop extrusion model proposes that a cohesin complex acts as an active motor extruding chromatin loops, whose anchor points along DNA are pairs of CTCF-binding sites with opposite orientation (left panel). Phase-separation-based models (such as the Strings&Binders) consider the scenario where a chromatin filament is represented as a polymer chain, with binding sites along the chain for cognate different diffusing molecular binders (right panel). (**b)** Viral infections can alter chromatin 3D structure. Polymer models, combined with experimental data (e.g. Hi-C [5]) and molecular dynamics simulations, provide ensembles of configurations describing chromatin structure under normal and virally infected conditions.

One popular model is based on the LE mechanism and posits that a molecular complex, assumed to be made of cohesin or condensin, acts as an active motor that binds to the DNA and actively extrudes a chromatin loop until it encounters specific blocking sites, thought to be CTCF-binding sites (i.e., CCCTC-binding factors) with opposite orientations (Figure 1a, left panel). Once the loop is fully extruded, the extrusion complex may eventually dissociate from the DNA filament, releasing the loop [12,13,18–22].

Another relevant mechanism known to shape chromatin organization is PS [23], which is the key ingredient of the Strings&Binders [11] or the bridging-induced [24] models. Here, chromatin interactions are mediated by diffusing bridging molecules, such as transcription factors (TFs), which bind pairs or even hubs of DNA distal sites, hence producing DNA loops through equilibrium polymer thermodynamics mechanisms (Figure 1a, right panel) [10,11,15,23–37]. Typically, a chromatin filament is modeled as a self-avoiding chain of beads having specific binding sites for cognate, diffusing molecular binders [25,26,38]. In this scenario, DNA molecule interactions lead to chromatin structural changes through thermodynamic phase transitions, such as coil-to-globule or PS events, which naturally facilitate contact or segregation of specific distal DNA regions, like genes and their enhancers.

To study the structural changes induced by SARS-CoV-2 virus observed in experimental Hi-C data [5], polymer models in which PS and LE simultaneously act have been employed [9] (Figure 1b). In this way, it has been possible to investigate how the virus impacts the physical processes driving chromatin organization. By performing massive MD simulations, ensembles of 3D structures representing chromatin in SARS-CoV-2-infected and non-infected conditions have been generated and successfully explained with Hi-C data [5].

### Modeling of chromatin re-structuring in A/B compartments upon SARS-CoV-2 infection

A significant structural re-arrangement in chromatin architecture due to SARS-CoV-2 infection occurs at the A/B compartment level [5]. Specifically, viral infection weakens the A-compartment and increases A/B compartment mixing. To quantitatively investigate this effect, a simple block copolymer model, where A and B compartments are schematized as two different types of binding sites (represented with different colors), has been used. Interactions are mediated by binders through homo-typic (E_A-A_ and E_B-B_) and hetero-typic (E_A-B_) affinities, with obviously E_A-A_ > E_A-B_ and E_B-B_ > E_A-B_ [39] to ensure A and B PS [10,29,39,40]. In [9], balanced (i.e., E_B-B_ = E_A-A_) and unbalanced (i.e., E_B-B_ > E_A-A_) interactions have been considered as control parameters of the model structural regime.

**Figure 2:**
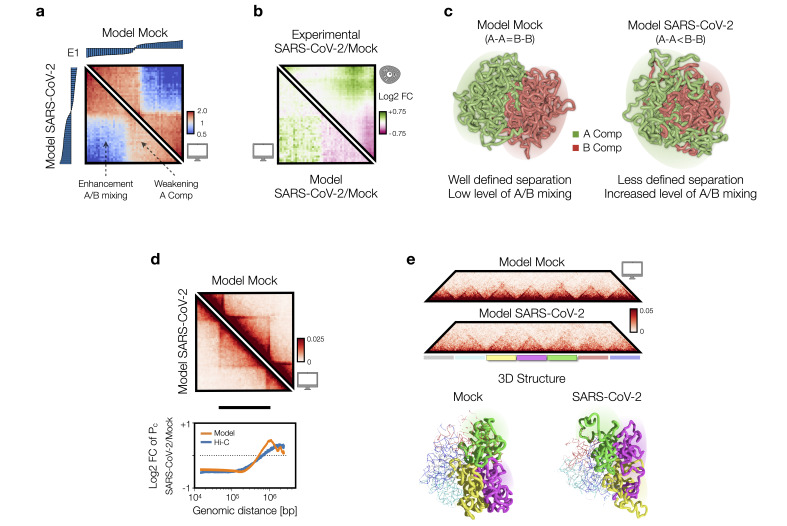
Modeling of chromatin re-structuring in A/B compartments and at TAD level. **(a)** Saddle plots of the best-fit polymer models for Mock (top right) and SARS-CoV-2 (bottom left) conditions. The sorted 1st eigenvector (**E1**) is displayed above for Mock and on the left for SARS-CoV-2. (**b)** Log2 fold change (FC) of the saddle plots from the model (bottom left) and Hi-C data [5] (top right). Pearson correlation between the model and experimental data is displayed. (**c)** Examples of 3D configurations of A/B compartments for Mock (left) and SARS-CoV-2-infected (right) conditions. (**d)** Average model contact maps of TADs for Mock (top right) and SARS-CoV-2-infected (bottom left) conditions, derived by fitting experimental contact probabilities. Below, the Log2 FC of contact probabilities in experimental Hi-C [5] (blue curve) and best-fit models (orange curve) is displayed. (**e)** Contact maps from the best-fit models for Mock (top) and SARS-CoV-2 (bottom) conditions. Below, 3D visualizations of polymer structures generated from MD simulations of the Mock (left) and SARS-CoV-2 (right) models. For clarity, only the three central TADs are displayed. Panels adapted from [9]. Abbreviations: MD, molecular dynamics; TADs, topologically associated domains.

From the contact maps computed *in silico*, the first eigenvector E1 of the principal component analysis and saddle plots of the ordered components of the eigenvector [41,42] have been computed and compared with experimental saddle plots from Hi-C data [5] (Figure 2a). Simple combinations of models with balanced and unbalanced interactions were able to accurately fit the change of saddle plots from Mock (i.e. control condition) to SARS-CoV-2-infected condition (Figure 2a). The best combination of interactions to reproduce the average compartment profile obtained from Hi-C data in Mock cells is mainly (90%) described by a model with balanced homo-typic interactions, suggesting similarity between A and B compartments. In contrast, SARS-CoV-2-infected cells data have been better described by a combination of unbalanced homo-typic interactions with E_B-B_ > E_A-A_, in agreement with the overall weakening of the A compartment and the increased A/B mixing. In addition, comparison of saddle plot Log2 fold change (SARS-CoV-2/Mock) matrices revealed a high level of agreement with experimental data (Pearson r = 0.77, Figure 2b) [9]. Similar results are observed if combinations of distinct hetero-typic affinities E_A-B_ are considered, keeping fixed and balanced homo-typic interactions (i.e., E_B-B_ = E_A-A_). Indeed, SARS-CoV-2-infected data align more closely with a model having stronger hetero-typic affinities compared with the Mock condition, once again in agreement with the enhanced A/B mixing [9].

The above discussed results indicate that chromatin re-arrangements observed in infected host genome can be explained by a re-modulation of affinities which, in turn, affects the tendency of compartments to microphase-separate, as also shown by the 3D rendering of polymer structures representing A and B compartments in Mock (Figure 2c, left panel) and SARS-CoV-2-infected (Figure 2c, right panel) conditions.

## Modeling of chromatin re-structuring in TADs upon SARS-CoV-2 infection indicates alteration of physical mechanisms shaping 3D organization

Viral infection has been shown to weaken intra-TAD contacts [5] while slightly increasing inter-TAD interactions and reducing cohesin levels, indicating decreased LE activity. To test this hypothesis, a polymer physics model that integrates both LE and PS mechanisms has been employed [9].

In such a model, LE and PS mechanisms work together, and the chromatin contact patterns observed in Hi-C data [5] result from their interplay. By adjusting the key parameters of the system, namely the interaction affinity and the average distance between the extruders (or equivalently their number on the polymer), different polymer populations were originated, and the relative contact maps and contact probability profiles were created and compared with contact probabilities profiles computed from Hi-C data in Mock and infected conditions [9].

The best model for Mock data returned an average distance between extruders of approximately 100–150 kbp, aligning with previous estimates from other Hi-C datasets [13]. In contrast, the model fitting the SARS-CoV-2-infected Hi-C data indicated a significantly reduced number of extruders, approximately halved, which corresponds with experimental findings of a genome-wide decrease in cohesin levels [5] due to viral infection (Figure 2d). Importantly, the analysis has further revealed that to accurately fit Hi-C data in SARS-CoV-2-infected cells, a reduction in extruders must be paired with a (15–20)% decrease in the interaction affinity between binders and chromatin. This dual reduction iaffects chromatin spatial colocalization and contributes to the observed weakening of intra-TAD contacts in the infected genomes, in strong agreement with experimental data (Figure 2d, bottom panel, Pearson r = 0.82).

Interestingly, the same analysis conducted on Hi-C data from cells infected with the human coronavirus HCoV-OC43 [5] returned a best model closely resembled the Mock case, with the same affinity and only a slight increase in the average distance between extruders (similar to SARS-CoV-2, but less pronounced), in full agreement with the experimental reports [5,43]. This suggested that the chromatin re-arrangements, driven by altered PS properties, are specific to SARS-CoV-2 and do not occur with other viruses. Using MD simulations, 3D structures representing the average TADs profile in Mock (Figure 2e, bottom left panel) and SARS-CoV-2-infected (Figure 2e, bottom right panel) conditions have been generated. These models effectively summarized the architectural re-arrangements within and between TADs post-infection.

Overall, those simulations showed that SARS-CoV-2 infection specifically alters genome organization by modifying essential physical mechanisms, such as LE and PS, which are crucial for chromatin structure.

### A real case of study: structural re-arrangements of IFN response *DDX58* locus

**Figure 3:**
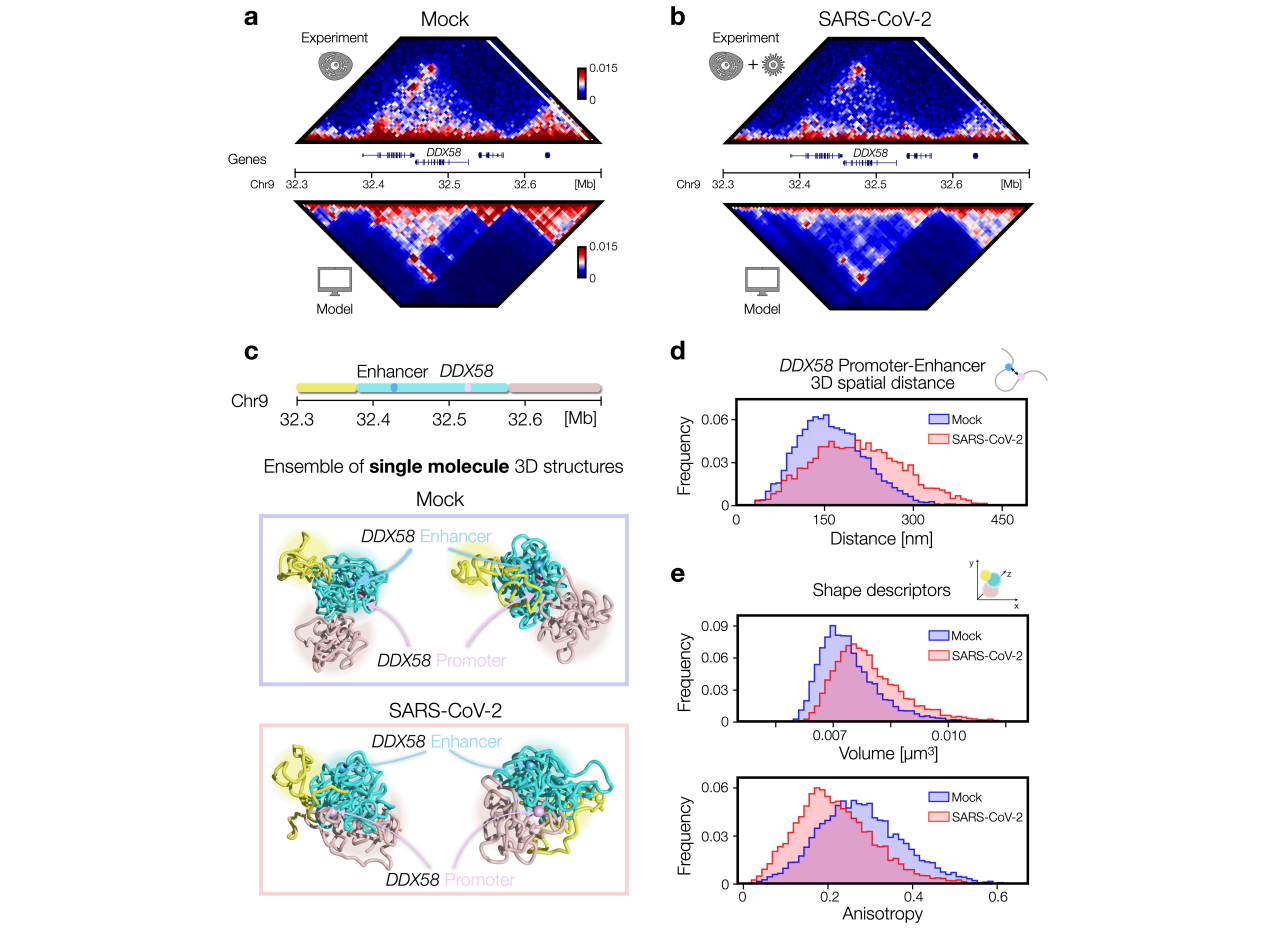
Structural re-arrangements of the IFN *DDX58* locus. **(a)** Hi-C data (from [5]) of the genomic region (chr9:32300000–32700000, hg19) centered on the IFN response, along with 3D structures obtained from MD simulations (bottom panel). (**b)** Hi-C data for the SARS-CoV-2-infected condition of the same genomic region. (**c)** Illustrations of 3D structures from the ensemble of single molecule configurations for the *DDX58* locus in Mock (top panel) and SARS-CoV-2-infected (bottom panel) models. The DDX58 promoter and its enhancer are highlighted in pink and cyan, respectively. (**d)** Distributions of 3D distances between the *DDX58* promoter and its enhancer, estimated from the population of simulated 3D structures. (**e)** Volume and anisotropy shape descriptors of the polymer representing the *DDX58* locus. Panels adapted from [9]. Abbreviations: IFN, interferon; MD, molecular dynamics.

IFN response genes are typically up-regulated in response to viral infections [16] and have been shown to be poorly expressed [1,44] in severe COVID-19 cases, resulting in an alteration of the host cell’s immune response to the infection. In line with the above discussed results, polymer models have been successfully used to study chromatin re-arrangements of real loci containing IFN response genes. Specifically, a 400 kbp genomic region around the *DDX58* gene (chr9: 32300000–32700000 bp, hg19 assembly, Figure 3a and b) has been considered. In the Mock condition, the *DDX58* gene is contained within a well-defined domain bordered by convergent CTCF sites. However, following SARS-CoV-2 infection, there is a noticeable weakening of intra-TAD interactions, although the CTCF peaks remain largely unchanged [5]. Similar findings were observed for other IFN loci, such as the *IFIT* gene cluster [5,9].

To quantitatively investigate these re-arrangements, a polymer model incorporating both LE and specific chromatin–protein interactions [23] has been employed. CTCF ChIP-seq data have been used to determine the probabilities and positions for extruder anchor points [13] and Hi-C data to optimize binding site types and their locations [9,15], as done in similar, recent studies [14,45]. Then, by means of MD simulations, populations of 3D structures have been generated and these accurately depict the differences in the *DDX58* locus between Mock and SARS-CoV-2 conditions (Figure 3a and b, lower panels). The simulated contact maps closely matched experimental data (Figure 3a and b, top panels, Pearson r > 0.9, average distance-corrected r’ = 0.67) [9]. In addition, rendering of snapshots from MD simulations, representing single-molecule 3D structures [14], visually showed the discussed architectural differences: in the Mock condition, the *DDX58* locus is organized in distinct, well-defined regions, whereas in SARS-CoV-2-infected cells, this locus appears less localized and more intermingled (Figure 3c).

Importantly, the population of 3D structures generated in Mock and SARS-CoV-2-infected cells allowed to study these architectural differences at the single-molecule level. Polymer models are particularly useful for this purpose, as they enable the creation of ensembles of independent 3D structures that mimic single-cell variability [23] and have been validated with experimental data from methods like MERFISH microscopy [46]. Therefore, the distributions of a number of physical observables can be calculated from the 3D structures in both Mock and SARS-CoV-2 (Figure 3d and e) models and quantitatively compared. An important example is the 3D distance between the *DDX58* promoter and its validated enhancer [5] (indicated on polymer structures in Figure 3c). Visual inspection of the 3D conformations revealed that in Mock conditions, the *DDX58* promoter and enhancer are closer in space compared with the infected condition, consistent with Hi-C data [5]. The distributions of 3D distances between the *DDX58* promoter and its enhancer (Figure 3d) supported this behavior, with the Mock condition showing a significant lower mean distance than the infected case [9]. Additionally, the distribution was more variable in infected cells (standard deviation in SARS-CoV-2 ∼30% higher than in Mock), indicating that gene mis-regulation upon infection is associated with a loss of contact specificity. This supported the idea that viral action alters the binding pattern through changes in cohesin and other factors, leading to a general loss of structural coherence in the population of 3D structures [17].

Analogously, other quantities describing the structure of the entire locus can be considered, such as polymer size and shape descriptors [47]. For example, distributions of volume and anisotropy have been estimated. It results that the volume distribution was more variable in SARS-CoV-2-infected cells (Figure 3e, top panel), with a standard deviation ∼30% higher compared with Mock. In contrast, the average anisotropy distribution (Figure 3e, bottom panel), which measures how asymmetrically the polymer is distributed in space, was lower in the SARS-CoV-2 population. These findings are consistent with previous observations of increased inter-TAD contacts and reduced localization, which in turn gives a strong indication that viral infection induces the loss of specificity of the host genome structure.

## Effects of other viral infections on host chromatin 3D structure

The above-described results show that experimental data for chromatin conformation of SARS-CoV-2-infected cells provide insights into the mechanisms underlying viral action on host genome, and it is helpful to develop accurate and predictive polymer physics models describing chromatin conformation in the infected cell. Notably, it is worth mentioning that this computational approach is absolutely general and can be employed, in principle, to investigate other viral infections. Indeed, other viruses, different from SARS-CoV-2, modify host chromatin architecture [48].

In general, by altering host chromatin organization, viruses can influence both viral and host gene expression, which is fundamental for their replication, latency, and pathogenicity [48]. However, not all viruses are able to modify host chromatin 3D organization in the same way. In fact, DNA and RNA viruses employ distinct yet overlapping strategies to achieve these effects. DNA viruses, such as herpesviruses and papillomaviruses, often persist in the host as chromatinized episomes, using host chromatin organizer proteins like CTCF and cohesin to regulate their gene expression. For instance, herpesvirus genomes adopt a circular episomal structure and recruit host chromatin machinery to regulate gene expression during latency and reactivation. In Epstein−Barr virus (EBV), chromatin loops mediated by CTCF and cohesin control the expression of essential latent genes such as *EBNA2*, as well as host oncogenes like *MYC*. This looping ensures a dynamic regulatory interplay between viral and host genomes, enabling the virus to establish and maintain latency while controlling viral reproduction activation [49–51]. Another example of DNA virus able to modify host chromatin organization is human papillomavirus, which regulates its oncogenes through interactions with host architectural proteins. CTCF binding within the viral genome suppresses the expression of the oncogenes *E6*/*E7*, which drive tumorigenesis. Disruption of these CTCF-binding sites leads to increased *E6*/*E7* expression and uncontrolled cell proliferation [52]. Viral integration into the host genome further alters chromatin structure by inducing structural variants and breaking TADs boundaries. These changes redirect host enhancers to viral oncogenes or create viral-host hybrid super-enhancers, which amplify oncogenic expression [52].

On the other hand, RNA viruses (like SARS-CoV-2), though typically not integrating into host DNA, can still profoundly alter host chromatin architecture through protein-mediated mechanisms. Specifically, human immunodeficiency virus (HIV) preferentially integrates into transcriptionally active euchromatin, near enhancers and TAD boundaries. The chromatin state of the integration site determines whether the virus remains latent or transcriptionally active. Furthermore, integrated HIV disrupts local chromatin organization, enabling viral gene expression while potentially silencing nearby host genes [53]. Finally, the influenza virus modulates host chromatin through multiple strategies. In particular, the influenza non-structural protein prevents transcription termination, disrupting cohesin-bound chromatin loops and increasing TFs accessibility. Simultaneously, the influenza virus nucleo-protein redistributes cohesin to specific loci, forming active chromatin loops that enhance viral replication [54,55].

## Conclusions

Recent experimental evidence highlighted that viral infections can alter chromatin 3D architecture of the host cell [3], with an impact on gene activity [4,5,48]. Notably, for the SARS-CoV-2 virus, such structural changes can occur at different scales within the 3D topology of the host genome and alter structural features of chromatin, from A/B compartments to gene-enhancer looping [9]. In this review, we discussed recent applications of polymer models to study how SARS-CoV-2 viral infection affects the multiscale 3D organization of chromatin in host cells, spanning from a few kbp to several Mbp and encompassing various structural elements such as A/B compartments, TADs, and gene-enhancer loops. To this aim, polymer physics models and MD simulations, commonly applied in the field of chromatin organization [11,24,25], have been used. A simple block copolymer model [10,29,39,40], incorporating homo-typic and hetero-typic interactions, can effectively describe the weakening of the A compartment and the increased mixing of A and B compartments observed in Hi-C data from SARS-CoV-2-infected cells by adjusting A-A affinities in an affinity-unbalanced A/B compartment model. On lower length scales, at the TAD level, the model suggests that a combined reduction in LE activity (reduction in extruders), together with altered PS properties (reduction in chromatin–protein affinities), quantitatively explains the weakened intra-TAD interactions observed in Hi-C data. Notably, this is not observed in the infection from the virus of common flu HCoV-OC43 [5,9], suggesting features typical of SARS-CoV-2. More complex models could potentially include additional features like nuclear envelope [39,56–58] interactions, which also influence chromatin structure, as well as PS of molecules interacting with chromatin [58].

Furthermore, polymer models informed with Hi-C and ChIP-seq data [5,9] of genomic loci containing genes relevant to host cell response to the infection, such as *DDX58* and *IFIT*, revealed greater variability in the ensemble of single-molecule conformations in SARS-CoV-2 infected condition. This potentially suggests the link between chromatin re-arrangements and the mis-regulation of antiviral response genes (IFN genes) during SARS-CoV-2 infection. Furthermore, given the growing evidence that viral infections, beyond SARS-CoV-2, alter host chromatin architecture, the same computational approaches, integrating models and data, can be used to understand the action of other viruses.

In the future, to comprehend the molecular causes underlying the observed chromatin re-arrangements, it would be useful to build models in which viral-specific factors are incorporated [2,59]. Indeed, employing polymer models in combination with experimental data can help assess the effects of specific proteins on chromatin architecture, promoting the identification of molecular targets for, e.g. therapeutic purposes. In general, the study of the link between viral infection and chromatin architecture provides valuable insights into how viruses influence host gene regulation. Polymer models are useful tools for this purpose, as well as phase transitions theory and complex emergent behaviors, common to biological systems and systems of soft-matter physics [60–74], as they offer an objective and predictive approach to linking various aspects of genome organization and function [75,76].

**Perspectives**

- Genome architecture is closely related to essential biological functions. Computational approaches based on polymer physics are valuable tools to quantitatively investigate the link between genome structure and activity. As some viral infections, such as SARS-CoV-2, are able to re-structure chromatin organization of the host cell, polymer models can be employed to understand the impact of the infection on the physical mechanisms shaping chromatin in space.
- Polymer models have been successfully used to model multiscale chromatin re-organization upon SARS-CoV-2 infection, from the A/B compartment level to the TADs scale and also regulatory enhancer−gene loops. Models suggest that genome re-arrangements are due to alteration of loop-extrusion and phase-separation properties of chromatin, leading to a loss of structural specificity and an altered activity of the genes involved in immunological response.
- Future research should continue leveraging polymer physics models to explore the specific viral proteins and host pathways involved in SARS-CoV-2-mediated chromatin re-organization and, analogously, for other viral infections. Incorporating more biologically accurate parameters into these models, such as viral protein interactions and specific chromatin states, will enhance the predictive power of the simulations.

## Open Access

This article has been published open access under our Subscribe to Open programme, made possible through the support of our subscribing institutions, learn more here: https://portlandpress.com/pages/open_access_options_and_prices#conditional

## Abbreviations

IFN: interferon
LE: loop extrusion
MD: molecular dynamics
PS: phase separation
TADs: topologically associated domains
